# Editorial on Cerebral endothelial and glial cells are more than bricks in the Great Wall of the brain: insights into the way the blood-brain barrier actually works (celebrating the centenary of Goldman's experiments)

**DOI:** 10.3389/fncel.2015.00128

**Published:** 2015-04-01

**Authors:** Elena García-Martín, George E. Barreto, José A. G. Agúndez, Rubem C. A. Guedes, Ramon S. El-Bachá

**Affiliations:** ^1^Department of Biochemistry, Molecular Biology, and Genetics, University of ExtremaduraCáceres, Spain; ^2^Departamento de Nutrición y Bioquímica, Facultad de Ciencias, Pontificia Universidad JaverianaBogotá, Colombia; ^3^Department of Pharmacology, University of ExtremaduraCáceres, Spain; ^4^Departamento de Nutrição, Universidade Federal de PernambucoRecife, Brazil; ^5^Laboratory of Neurochemistry and Cell Biology, Department of Biochemistry and Biophysics, Health Sciences Institute, Federal University of BahiaSalvador, Brazil

**Keywords:** blood-brain barrier, glial cells, cerebral endothelial cells, xenobiotic-metabolizing enzymes, ABC transporters, oxidative stress response, neuroinflammation

In August 23 (1913), The Lancet reported the death of Dr. Edwin Ellen Goldmann in its obituary, which had occurred on August 12th of this same year due to a malignant disease of the liver. The introduction of methylene blue as a histological reagent by Paul Ehrlich led to the discovery that all organs, but the brain, were stained after its injection into the blood stream. Goldmann showed the inverse by staining only the brain after injection of dyes into the cerebro-spinal fluid. Concerning the work of Dr. Goldmann, Dr. F. W. Mott wrote in the obituary of The Lancet (1913): “We have learnt from his work that the cerebro-spinal fluid receives from the choroid plexus important products which are carried to the nervous system tissue. Also the plexus protects the nervous tissue from the penetration of toxic substances.” A century after his pioneering work, which supported the idea of the existence of a blood-brain barrier (BBB), we decided to celebrate it by proposing this Research Topic to Editors of Frontiers in Cellular Neuroscience.

We would like to thank all contributors for their valuable work helping us to present wide-ranged aspects in the field. Currently we know that a physical barrier due to the formation of tight junctions in cerebrovascular endothelial cells is the main component of the BBB. In this Research Topic, the implication of astrocyte functions in the protection of the BBB was reviewed (Cabezas et al., 2014). One year after the Goldmann's death, the First World War began in Europe leading to the death of 16 million people due to the military industry technological sophistication, which has increased since then and continues to kill several human lives. An article reviewing alterations in the brain milieu causing dysfunction or disruption of the BBB following exposure to blast shock waves was published in this Topic (Shetty et al., 2014). Since cerebrovascular diseases are prevalent worldwide, affecting the structure and functions of the BBB, cellular effectors to recover the neurovascular unity integrity were reviewed (Posada-Duque et al., 2014).

Currently we know that the BBB is more than a physical barrier. Several drug- and xenobiotic-metabolizing enzymes are expressed in endothelial and glial cells, constituting a metabolic barrier. The expression of xenobiotic metabolizing enzymes in the BBB was discussed (Agúndez et al., 2014). However, the attempt to protect neurons from xenobiotic by metabolizing them sometimes fails. Catechol is a compound that induces glutathione (GSH) depletion, which leads to apoptosis (Lima et al., 2008). This depletion is due to glutathione transferases (GST; EC 2.5.1.18), which catalyzes the conjugation of catechols to GSH. Therefore, the inhibition of xenobiotic metabolizing enzymes is sometimes useful to protect neurons. The inhibitory effect of 8-methoxypsoralen on GST-π activity was investigated (Oliveira et al., 2014). Although the glucuronidation of catechols was not catalyzed by brain microsomes, planar phenols could be conjugated (El-Bachá et al., 2000). The importance of UDP-glucuronosyltransferases (EC 2.4.1.17) in the BBB was reviewed (Ouzzine et al., 2014). ABC-transporters in endothelial and glial cells exert an active function in the BBB. An *in vitro* stroke model of the BBB was used to investigate how oxygen/glucose deprivation can affect tight junction proteins, as well as the expression of ABC-transporters (Neuhaus et al., 2014).

It seems that there is also an antioxidant barrier, which protects the central nervous system against the oxidative stress. The endogenous protection against reactive oxygen species can occur through the increased expression of mitochondrial enzymes in astrocytes (Cabezas et al., 2012). The expression of heme oxygenase (EC 1.14.99.3) isoform 1 (HMOX1) is modulated by pro-oxidants in neuroglia. In another study, an association between HMOX1 genetic variants and Parkinson's disease was investigated (Ayuso et al., 2014). Furthermore, biomarkers of this disease were investigated on cerebrospinal fluid of patients (Jiménez-Jiménez et al., 2014).

Cells involved in the BBB can establish immune communications. Interactions between the microglia and the vascular system (Fonseca et al., 2014), as well as the role of macrophages at the brain borders, how they enter and differentiate inside the brain, and their functions in health and neuroinflammation were reviewed (Corraliza, 2014). The effect of inflammatory stimulus on neuron/glial co-cultures infected with *N. caninum* was evaluated (Jesus et al., 2014).

The connection between the BBB and pain was reviewed, focusing on cellular and molecular mechanisms of BBB permeability induced by inflammatory or neuropathic pain and migraine (DosSantos et al., 2014). The BBB in gliomas was also reviewed (Dubois et al., 2014).

A century after the work of Goldmann, who contributed to the discovery of the BBB, several questions remains without answers. However, it is already known that BBB is far more than a physical barrier. Endothelial and glial cells also exert metabolic, antioxidant and immunological activities. It seems of great importance to understand the role of BBB in neurodegenerative diseases. Compounds that could show an ability to repair BBB injury, to suppress neuroinflammation and to provide neuroprotection remain to be discovered. The role of microglia and macrophages in the BBB needs to be better understood, if we want to design effective therapies against neuroinflammation. Efforts to discover strategies that could protect neurons in ischemic events continue to defy neuroscientists. Advances in biochemistry, molecular biology, and genetics will improve our knowledge about the BBB in the next 100 years.

## Conflict of interest statement

The authors declare that the research was conducted in the absence of any commercial or financial relationships that could be construed as a potential conflict of interest.
